# Quality of life in relation to tamoxifen or exemestane treatment in postmenopausal breast cancer patients: a Tamoxifen Exemestane Adjuvant Multinational (TEAM) Trial side study

**DOI:** 10.1007/s10549-012-2028-2

**Published:** 2012-03-28

**Authors:** J. G. H. van Nes, D. B. Y. Fontein, E. T. M. Hille, D. W. Voskuil, F. E. van Leeuwen, J. C. J. M. de Haes, H. Putter, C. Seynaeve, J. W. R. Nortier, C. J. H. van de Velde

**Affiliations:** 1 Department of Surgery, K6-R, Leiden University Medical Centre, P.O. Box 9600, 2300 RC Leiden, The Netherlands; 2 Department of Epidemiology, Netherlands Cancer Institute, Amsterdam, The Netherlands; 3 Department of Psychology, Academic Medical Center, University of Amsterdam, Amsterdam, The Netherlands; 4 Department of Medical Statistics, Leiden University Medical Centre, Leiden, The Netherlands; 5 Department of Radiotherapy, University Medical Center Groningen, Groningen, The Netherlands; 6 Department of Medical Oncology, Erasmus MC–Daniel den Hoed Cancer Center, Rotterdam, The Netherlands; 7 Department of Medical Oncology, Leiden University Medical Centre, Leiden, The Netherlands

**Keywords:** Adverse effects, Breast cancer, Exemestane, Quality of life, Tamoxifen

## Abstract

Tamoxifen and aromatase inhibitors are associated with side effects which can significantly impact quality of life (QoL). We assessed QoL in the Tamoxifen Exemestane Adjuvant Multinational (TEAM) Trial and compared these data with reported adverse events in the main database. 2,754 Dutch postmenopausal early breast cancer patients were randomized between 5 years of exemestane, or tamoxifen (2.5–3 years) followed by exemestane (2.5–2 years). 742 patients were invited to participate in the QoL side study and complete questionnaires at 1 (T1) and 2 (T2) years after start of endocrine treatment. Questionnaires comprised the EORTC QLQ-C30 and BR23 questionnaires, supplemented with FACT-ES questions. 543 patients completed questionnaires at T1 and 454 patients (84 %) at T2. Overall QoL and most functioning scales improved over time. The only clinically relevant and statistically significant difference between treatment types concerned insomnia; exemestane-treated patients reported more insomnia than tamoxifen-treated patients. Discrepancy was observed between QoL issue scores reported by the patients and adverse events reported by physicians. Certain QoL issues are treatment- and/or time-specific and deserve attention by health care providers. There is a need for careful inquiry into QoL issues by those prescribing endocrine treatment to optimize QoL and treatment adherence.

## Background

The majority of breast cancer patients are diagnosed at postmenopausal age and most have hormone receptor-positive tumors. Over time, adjuvant endocrine therapy has increasingly been used to reduce disease recurrence and improve survival [1]. Presently, optimal endocrine therapy consists of at least 5 years of treatment including an aromatase inhibitor (AI), either given upfront or as part of a sequential treatment regimen following tamoxifen [2]. Both regimens are appropriate treatment options for postmenopausal hormone receptor-positive breast cancer patients [2, 3]. However, many patients on endocrine therapy are confronted with adverse effects, which may negatively impact QoL, treatment compliance, and may then lead to a reduced survival [4, 5]. The impact of long-term endocrine treatment on quality of life (QoL) in postmenopausal breast cancer patients may therefore be an important issue of deliberation regarding the choice for a specific adjuvant treatment strategy.

Both tamoxifen, a selective estrogen receptor modulator, and AIs, which potently inhibit the aromatase enzyme (involved in the conversion of androgens to estrogen), are associated with a variety of adverse effects. Tamoxifen is associated with thromboembolic complications and endometrial cancer while AIs show fewer life-threatening side effects but more readily give rise to sometimes invalidating symptoms such as hot flashes, arthralgias, vaginal dryness, and osteoporosis [6, 7]. Variations in the types and severities of adverse effects associated with the use of either tamoxifen or an AI may result in differences in the domains of QoL affected in patients using either endocrine treatment.

So far, several trials have investigated QoL in patients using adjuvant endocrine therapy, but only four have compared QoL in patients treated with tamoxifen versus an AI [8–12]. It is difficult to compare these studies due to variations in trial design, starting time of the AI, and type of AI used. To the best of our knowledge, the ATAC QoL study is the only large trial that compared QoL from the start of endocrine therapy in patients treated with tamoxifen versus an AI upfront [9]. In the Tamoxifen Exemestane Adjuvant Multinational (TEAM) trial (Netherlands Trial Register NTR267), postmenopausal, hormone receptor-positive early breast cancer patients were randomized to either 5 years of exemestane upfront or 2.5–3 years tamoxifen followed by 2–2.5 years of exemestane [2]. There was a major participation in the TEAM study from the different hospitals throughout the Netherlands, therefore, this study provided a good opportunity for studying the effects of exemestane and tamoxifen on QoL in a homogeneous cohort of Dutch breast cancer patients. Moreover, we were able to relate relevant QoL issues reported by patients in this side study to the adverse events involved with these issues reported by the same patients in the main study using the registered adverse events.

## Patients and methods

### Study design

The study design and patient eligibility criteria for the TEAM trial have been described previously [2]. In the Netherlands, the study was initiated in 76 hospitals and details also have been described previously [13]. The TEAM QoL side study was an open multicenter study in which 45 Dutch TEAM centers participated. The side study protocol was approved separately by central and local ethics authorities before the enrollment of patients.

### Patients and data collection

Patients who were randomized between January 2nd, 2003 and December 29th, 2004 and were event-free were invited to participate in the TEAM QoL side study. Patients received a letter together with the first QoL questionnaire at 1 year after treatment randomization (further referred to as time point 1; T1). Participating patients who returned the first questionnaire and were disease-free 2 years after randomization received the second questionnaire 1 year after T1 (further referred to as time point 2; T2). Patients included in the sequential arm received the second questionnaire before the switch from tamoxifen to exemestane. No questionnaire was sent at baseline (time of diagnosis and treatment) as the results regarding QoL may potentially be biased, due to the recent knowledge of breast cancer diagnosis and impending treatment, which is known to have a negative impact on QoL. Furthermore, treatment was allocated by randomization, hence there is essentially no indication for baseline imbalance in QoL data between both treatment arms [14]. Patient, tumor, treatment, and survival data were collected through the main TEAM Datacenter in Leiden, the Netherlands. In the main trial, patients were seen every 3 months in the first year, twice yearly in the second year and at least yearly thereafter. In the main trial, data on adverse events experienced by patients were recorded during follow-up visits by local investigators and centrally collected at the main datacenter. For the QoL participants, we selected adverse events reported within the first 2 years that were associated with the relevant QoL issues observed from the central database.

### Questionnaires

Data on QoL were obtained using the European Organization for Research and Treatment of Cancer Quality of Life Questionnaire Version 3.0 (EORTC QLQ-C30) and the EORTC Breast Cancer Module questionnaire (QLQ-BR23), both translated into Dutch and previously validated [15, 16]. Both questionnaires were used after authorization by the EORTC Quality of Life Study Group. The EORTC QLQ-C30 is composed of five functioning scales (physical functioning, role functioning, cognitive functioning, emotional functioning, and social functioning), a global health status/QoL scale, three symptom scales (fatigue, pain, nausea/vomiting), and six single items (dyspnea, appetite loss, sleep disturbance, constipation, diarrhea, and financial impact). The EORTC QLQ-BR23 is a validated tool designed for breast cancer patients with varying disease stages and treatment modalities and consists of 23 items that assess disease symptoms, side effects, body image, sexual functioning, future perspectives, therapy side effects, breast and arm symptoms, and hair loss. Items that specifically assess side effects of chemotherapy were not applicable for the current study. In addition, the Functional Assessment of Cancer Therapy-Endocrine Subscale (FACT-ES) questionnaire was designed and validated to measure QoL in breast cancer patients treated with endocrine therapies [17]. Of the 18 items, 13 were included in our questionnaire (as other items were already included through the EORTC QLQ-C30 or BR23 questionnaires), resulting in three endocrine symptom scales (menopausal complaints, weight complaints, and vaginal complaints).

Based on standard EORTC scoring procedures, all scales were linearly converted to a 0-to-100 scale. Missing data were treated according to published recommendations [18]. For scales evaluating global health and functioning, higher scores represent higher levels of functioning and health status. For the evaluation of symptoms, higher scores correspond to more problems and higher levels of complaints.

### Relevant patient-reported outcomes

Regarding QoL, the following items were investigated: (1) the difference between the QoL scores for patients using tamoxifen versus exemestane, (2) the difference between the two time points (T1 and T2), and (3) the interaction between treatment arm and time. A difference in score of at least eight points between groups was considered clinically relevant, and has been demonstrated to be a reasonable cut-off for clinical significance for a range of QoL endpoints [19]. Prior surgery was taken into account for analyses of body image, sexual functioning, and sexual enjoyment.

To study the association between the relevant QoL issues as reported by the patients and the related adverse events recorded for these patients by their treating physicians in the main database, patients whose questionnaire item scores were worse than the mean EORTC QLQ-C30 and BR23 reference scores were considered for comparison [20].

### Statistical analysis

All data were analyzed using the statistical package SPSS for Windows 17.0 (SPSS Inc, Chicago, IL, USA). Descriptive data are given as mean (SD) or median (range). The *t* test was used to compare frequencies between groups. Linear mixed models were used to assess changes over time for overall QoL and for separate components of QoL.

## Results

### Demographics

**Fig. 1:**
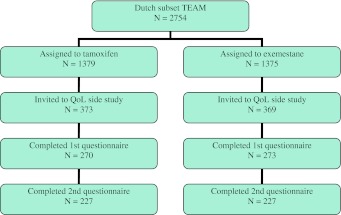
Patient selection

A total of 742 Dutch patients were invited to participate in the QoL side study (Fig. 1). Five-hundred-forty-three patients (73 %) completed the first questionnaire, of which 454 (84 %) also completed the second questionnaire. Baseline characteristics of the responding patients and the total group of Dutch TEAM patients are shown in Table 1. The distribution of clinicopathological and treatment characteristics of patients participating in the QoL side study was similar to that of the entire cohort of Dutch TEAM trial patients, except for the distribution of age, hormone receptor status, and prior chemotherapy (yes/no). Of the patients participating in the TEAM QoL side study, most were older than 60 years, had node-positive disease, and underwent a sentinel lymph node procedure followed by an axillary lymph node dissection. Almost 50 % of the tumors were smaller than 20 mm and approximately half of the patients were treated by mastectomy.

**Table 1:** Clinicopathological data of responders and all Dutch TEAM patients

	Responders		TEAM NL		*p*-value
	*N*	%	*N*	%	
Total	543	100	2,753	100	
Age					
<50–59	200	37	914	33	0.039
60–69	200	37	965	35	
≥70	143	26	874	32	
Body mass index					
≤25	190	39	919	38	0.589
25–30	188	39	931	38	
≥30	109	22	601	25	
Pathological tumor stage					
T1	267	49	1,235	45	0.158
T2	241	45	1,329	48	
T3 and T4	32	6	183	7	
Pathological nodal stage					
pN0	150	29	834	31	0.166
pN1–3	275	53	1,387	52	
pN4–9	77	15	327	12	
pN ≥ 10	18	3	131	5	
Histological grade					
Grade I	85	17	420	16	0.896
Grade II	244	48	1,218	47	
Grade III	179	35	934	36	
Type of tumor					
Ductal	404	75	2,047	75	0.891
Lobular	84	16	442	16	
Ductal lobular	27	5	129	5	
Other	21	4	109	4	
Hormone receptor					
ER+, PgR+	350	64	1,950	71	0.001
ER+, PgR−	129	24	595	22	
ER+, PgRnp	54	10	153	6	
ER−, PgR+	10	2	47	2	
ER−, PgR−	0	0	6	0	
Local therapy					
MST, RT−	188	35	1,127	41	0.051
MST, RT+	92	17	401	15	
BCS, RT−	7	1	36	1	
BCS, RT+	255	47	1,188	43	
Treatment axilla					
SLNP−, ALND−	0	0	3	0	0.882
SLNP−, ALND+	172	32	885	32	
SLNP+, ALND−	127	23	632	23	
SLNP+, ALND+	244	45	1,233	45	
Chemotherapy					
No	348	64	1,941	71	0.002
Yes	195	36	812	30	

*ALND* axillary lymph node dissection; *BCS* breast conserving surgery; *ER* estrogen receptor; *MST* mastectomy; *np* not performed; *PgR* progesterone receptor; *RT* radiotherapy; *SLNP* sentinel lymph node procedure; *TEAM NL* all patients included in the Netherlands

### QoL: tamoxifen versus exemestane

**Fig. 2:**
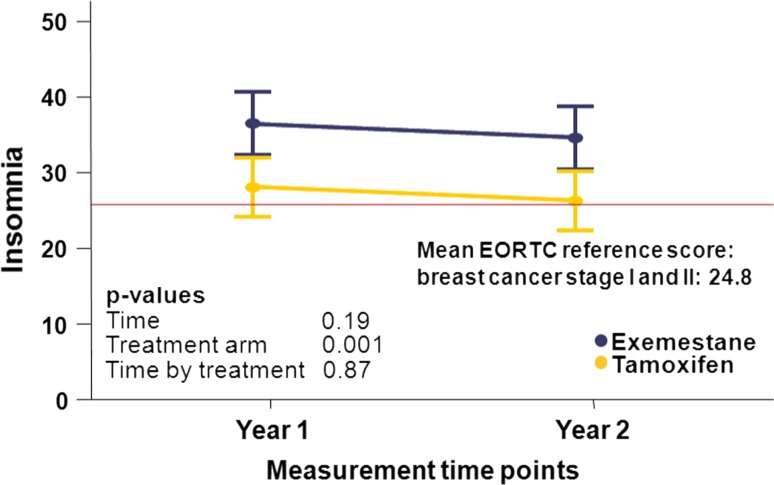
Insomnia in relation to treatment and time in the TEAM QoL side study

The results regarding QoL-items are shown in Table 2. In general, the scores for the various issues did not differ significantly between patients using tamoxifen versus exemestane. Patients allocated to tamoxifen showed superior scores for emotional functioning and sexual functioning (*p* = 0.048 and *p* = 0.024 respectively) than exemestane users. Treatment with exemestane did not show superior results compared to tamoxifen for any of the functioning scales. Regarding individual symptoms, patients who received tamoxifen had fewer complaints of fatigue, dyspnea, insomnia, and arm symptoms than patients receiving exemestane. For “fatigue,” the results were unrelated to the administration of chemotherapy (data not shown). Only for insomnia, the differences between the two treatment types were clinically significant (more than eight points difference between tamoxifen and exemestane), observed at both time points (Fig. 2). The endocrine symptom scales that were assessed using the FACT-ES included menopausal, weight, and vaginal complaints. These scores did not differ between treatment arms.

**Table 2:** Overview of the different functioning and symptom scales by time and treatment arm

	T1		T2		*p*-value		
	Tamoxifen	Exemestane	Tamoxifen	Exemestane	Treatment	Time	Time by treatment
	Mean (SD)	Mean (SD)	Mean (SD)	Mean (SD)			
EORTC QLQ-C30							
Functioning scales							
Physical functioning	80 (18)	78 (18)	79 (18)	79 (17)	0.732	0.508	0.132
Role functioning	80 (25)	79 (28)	82 (26)	82 (25)	0.741	0.028	0.826
Cognitive functioning	83 (21)	79 (25)	85 (19)	83 (22)	0.082	0.002	0.179
Emotional functioning	80 (21)	75 (21)	83 (20)	81 (21)	0.048	<0.001	0.273
Social functioning	87 (20)	86 (19)	90 (19)	90 (19)	0.861	0.001	0.397
Global health scale							
Global health status	78 (18)	75 (19)	78 (17)	76 (17)	0.074	0.458	0.281
Symptom scales							
Fatigue	30 (25)	34 (26)	24 (23)	29 (23)	0.026	<0.001	0.661
Pain	20 (24)	21 (25)	18 (24)	20 (25)	0.216	0.234	0.643
Nausea and vomiting	6 (15)	6 (17)	4 (14)	3 (14)	0.917	0.004	0.395
Symptom single items							
Dyspnea	15 (24)	20 (25)	14 (23)	18 (24)	0.032	0.234	0.490
Appetite loss	9 (22)	9 (19)	5 (16)	6 (15)	0.697	0.001	0.511
Insomnia	28 (32)	37 (31)	27 (30)	35 (31)	0.001	0.188	0.869
Constipation	12 (24)	12 (23)	13 (24)	10 (55)	0.319	0.507	0.337
Diarrhea	4 (15)	6 (17)	4 (15)	5 (15)	0.236	0.458	0.823
Financial difficulties	8 (18)	5 (15)	9 (22)	6 (16)	0.076	0.431	0.791
EORTC QLQ-B23							
Functioning scales							
Body image^a^	84 (25)	83 (22)	87 (20)	84 (26)	0.327	0.004	0.294
Sexual functioning^a^	20 (19)	17 (19)	21 (19)	16 (19)	0.024	0.755	0.208
Sexual enjoyment^a^	50 (30)	46 (23)	48 (22)	44 (22)	0.172	0.162	0.829
Future perspective	67 (27)	64 (26)	70 (25)	72 (25)	0.710	<0.001	0.028
Symptom scales							
Syst therapy side effect	19 (17)	19 (18)	17 (17)	18 (17)	0.964	0.040	0.426
Breast symptoms	22 (19)	19 (19)	16 (19)	14 (19)	0.152	<0.001	0.523
Arm symptoms^b^	19 (21)	17 (21)	20 (20)	16 (20)	0.027	0.728	0.213
EORTC FACT-ES							
Menopausal complaints	26 (21)	26 (21)	24 (20)	26 (20)	0.572	0.093	0.196
Weight complaints	14 (20)	16 (20)	15 (19)	16 (19)	0.186	0.207	0.972
Vaginal complaints	18 (27)	20 (27)	20 (26)	23 (26)	0.337	0.412	0.793

^a^For this analysis, surgery was a stratification factor: mastectomy versus breast conserving surgery

^b^For this analysis, surgery was a stratification factor: axillary lymph node dissection (no/yes)

The global health status scale represents an overall summary measurement of QoL. With respect to either treatment group, there was no difference in global health status/overall QoL (Table 2). Interestingly, the reported overall QoL was higher than the reference value of the EORTC QLQ-C30 (>75 vs. 62 points).

### QoL: changes over time

Changes in QoL items were assessed over the 1-year period between T2 and T1 for the total group of patients, as there were no relevant differences between the two treatment types. We found that over time, most functioning scales improved, except for physical functioning, sexual functioning, and sexual enjoyment (*p* < 0.01). Of note, fewer patients completed the questions concerning sexual functioning and enjoyment compared to the other items (data not shown). Over time, there was also no change in global health status; neither improvement nor deterioration. Concerning the individual symptom scales, a significant improvement was found for the following items: fatigue, nausea and vomiting, appetite, breast symptoms, and side effects of systemic therapy. Again, these results for fatigue were unrelated to the administration of chemotherapy (data not shown). A clinically significant difference over time was only established for breast symptoms.

### QoL: interaction between treatment arm and time

Irrespective of treatment, most assessed items improved from T1 to T2. Only for the functioning scale “Future perspective” did an interaction exist between treatment and time: patients using exemestane improved more compared to patients using tamoxifen.

### QoL compared to relevant adverse events issues reported in the TEAM trial

The QoL side study scores for sexual functioning and for sexual enjoyment were below the mean EORTC QLQ-C30 reference score for 58 % of patients and 72 % of patients, respectively, at T1; and values were similar at T2 [20]. In contrast, adverse events related to sexual functioning and/or sexual enjoyment from the central database, including genital or vaginal discharge, decreased/loss of libido, vaginal dryness, and vulvovaginal disorders, were only documented for 3 % of the QoL participants. Concerning insomnia, almost 60 % of the QoL patients had a higher score compared to the mean reference score of the EORTC at T1 and T2 (indicating more sleeping problems), while in the central database, insomnia was recorded as adverse event by only 4 % of the QoL study participants [20]. Lastly, fatigue was reported as adverse event by 12 % of the QoL participants in the main TEAM database compared to 45 % of QoL study patients having a higher score than the mean EORTC reference score for fatigue, indicating more complaints, observed at both T1 and T2 [20].

## Discussion

The impact of adjuvant endocrine therapy on QoL is an ongoing discussion in the treatment of breast cancer patients prescribed long-term endocrine therapy. The current standard of practice advocating 5 or more years of endocrine treatment can therefore be considered cumbersome in those experiencing severe adverse effects. Both tamoxifen and AIs have been associated with the development of various menopausal symptoms like sleeping disorders and sexual problems related to the depletion of circulating estrogens, some of which being severe to the point of significantly diminishing QoL. The present investigation of QoL in patients in the TEAM trial offers further insight into the impact of either tamoxifen or exemestane on a woman’s QoL during endocrine therapy for breast cancer.

In the present investigation, a clinically significant difference was found between the two treatment arms for insomnia, observed at both time points, indicating more problems for exemestane users versus those taking tamoxifen. In general, insomnia is underreported and frequently overlooked in the context of breast cancer treatment. Approximately, half of all breast cancer patients experience sleeping disorders up to several years post-diagnosis [21]. The pathophysiological mechanism behind insomnia in breast cancer patients suggests a relation with nocturnal hot flashes [22]. Both hot flashes and musculoskeletal symptoms have also been associated with the depletion of circulating estrogens [23]. As exemplified by the MA.17 trial, a significant increase in the incidence of hot flashes and musculoskeletal symptoms was found in patients treated with letrozole compared to placebo [11]. Our data regarding more sleeping disorders in exemestane users suggests that further lowering of postmenopausal estrogen levels with exemestane may lead to more sleeping disorders. Unfortunately, this cannot be verified with blood samples, as these were not collected for our cohort of TEAM patients.

Patients using exemestane reported less sexual enjoyment and more sexual functioning problems than patients using tamoxifen. This is similar to the results as found after 1 year of therapy in the US Oncology side study of the TEAM trial concerning menopausal symptoms [10]. Our data do show that also after 2–2.5 years of therapy, menopausal symptoms persisted over time. In physiological menopause, the lack of circulating estrogens reduces vaginal lubrication, resulting in vaginal dryness and, consequently, dyspareunia [24]. Tamoxifen affects sexual functioning in terms of decreased libido and the ability to become aroused and experience orgasm, while AIs cause vaginal dryness and dyspareunia. Although tamoxifen is known to have anti-estrogenic properties on breast tissue, it exerts an estrogen agonist effect on the female genital tract in postmenopausal women and increases the risk of endometrial cancer [25]. Furthermore, under tamoxifen treatment, the vaginal squamous epithelium is weakly stimulated and undergoes proliferation and maturation [26]. It is possible that the abovementioned reasons explain why sexual functioning may be less affected in tamoxifen-treated patients than in those treated with exemestane. Another contributing factor may be that as already said, exemestane induces further lowering of postmenopausal estrogens in breast cancer patients. Fewer reports investigated vaginal dryness and dyspareunia in studies with AIs, but Morales suggest that AIs induce more symptoms of vaginal atrophy (vaginal dryness and dyspareunia) than tamoxifen, which parallels our findings that exemestane-treated patients reported more sexual functioning problems than tamoxifen-treated patients [27].

### Adverse events and reported QoL

Although it is difficult to relate QoL issues as measured with questionnaires with adverse events as documented by the physician, we observed striking differences between these two methods. With respect to specific aspects of QoL such as sexual functioning, fatigue, and insomnia, significantly more patients reported complaints of these items in the QoL side study than that adverse events related to these specific complaints were documented in the main TEAM trial database. This finding reiterates the importance of thorough investigations on QoL issues and questions the reliability of the reported adverse events in large multinational phase III trials. Ideally, every large clinical trial assessing efficacy and safety of new oncological treatments should include a questionnaire-based QoL assessment, enabling more precise estimation of the associated adverse events.

### Other QoL studies

To date, only a few large randomized trials comparing adjuvant tamoxifen with an AI have reported on QoL data (Table 3) [8–12]. Also, it is difficult to compare the different randomized trials with each other and with our QoL side study, due to differences in patient populations, countries of residence, AIs used, timing and of start of treatment, and the instruments used to assess QoL. However, regardless of these variations, no large differences in QoL were seen between tamoxifen and AIs.

**Table 3:** Overview of other quality of life studies in trials comparing tamoxifen with an aromatase inhibitor in postmenopausal early breast cancer patients

Study	Main/QoL	Author	Intervention	Sample size	Follow-up	QoL instrument	Outcome QoL study	Insomnia
ATAC	Main	Howell et al. [28]	Arm 1: Ana Arm 2: Tam (Arm 3: Combi)	9,366	n.r.	None	AI: Fewer gyn and vascular disorders; more arthralgia and fractures than Tam	n.r.
ATAC	QoL	Fallowfield et al. [8]	Arm 1: Ana Arm 2: Tam (Arm 3: Combi)	1,021	Baseline, 3, 6, 12, 18, and 24 months	FACT-B + ES	No sign differences across both groups. AI: fewer cold sweats and vaginal discharge Tam: more vaginal dryness, painful intercourse and loss of sexual interest.	n.r.
IES	Main	Coombes et al. [29]	Tam ➾ Exe	4,724	Baseline, 3, 6, 9, 12, 18, and 24 months	None	Switching to Exe had no adverse effect on QoL Exe: Fewer gyn complications than Tam	Tam: 16.8 % Exe: 19.6 % *p* = 0.02
IES	QoL	Fallowfield et al. [9]	Tam ➾ Exe	582	Baseline, 3, 6, 9, 12, 18, 24, 30, 36, 48, and 60 months	FACT-B + ES	No differences in QoL or in the endocrine subscale	Tam: 34.5 %, Exe: 34.6 %
MA 17	Main	Goss et al. [30]	After 5 years of Tam: Plac 5 years Let 5 years	3,612	Baseline, 6 months and annually	None	Vaginal bleeding more common in placebo versus letrozole (6 % vs. 8 % *p* = 0.005)	Let: 6 % Plac: 5 % *p* = 0.06
MA.17	QoL	Whelan et al. [12]	After 5 years Tam: Plac 5 years Let 5 years	3,612	Baseline, 6 months and annually	SF-36 MENQOL	Small differences in bodily pain and vasomotor symptoms. No differences in overall QoL	n.r.
TEAM	Main	Van de Velde et al. [2]	Tam ➾ Exe versus Exe	9,779	Baseline, every 3 months in year 1, yearly thereafter	None	Sequential arm: more gyn/endometrial symptoms and venous thrombosis; Exe alone: more musculoskeletal effects, hypertension and hyperlipidemia	Tam: 10 % Exe: 13 % *p* = <0.001
TEAM	QoL	Jones et al. 2007 [10]	Tam ➾ Exe versus Exe	1,614	Baseline and every 3 months	Ten common symptoms	More sleeping disorders for exemestane	n.r.
TEAM	QoL	Current: Van Nes/Fontein et al.	Tam ➾ Exe versus Exe	543	After 1 and 2 years of start endocrine therapy	EORTC C30 EORTC B23 FACT-B-ES	No difference in overall QoL between both groups	Exe > Tam (*p* = 0.001)

*Ana* anastrozole; *combi* combination; *ES* endocrine symptom subscale; *Exe* exemestane; *FACT-B* functional assessment of cancer therapy-breast; *Let* letrozole; *MENQOL* menopause specific quality of life questionnaire; *n.r.* not reported; *Plac* placebo; *SF-36* short form 36-item health study; *Tam* tamoxifen; *Gyn* gynaecological

The planned nature of the QoL side study using validated questionnaires as well as the high response rate for both T1 and T2 questionnaires lends confidence to our findings. The absence of a baseline measurement may be considered a shortcoming when assessing changes in QoL over time; notably, however, baseline measurements of QoL are likely biased due to recent knowledge of breast cancer diagnosis in our patient population at the start of treatment. Due to the randomized nature of this trial, differences in baseline QoL with respect to treatment arms are unlikely [14]. This study is limited by the lack of reporting consistency of adverse events in the main TEAM trial in relation to the observed QoL domains affected in patients in the side study. Concurrently, this may still adequately reflect variations in reporting by both investigators and patients alike during clinical visits.

## Conclusion

Our findings indicate that overall QoL and most functioning scales improve with longer therapy duration, both for patients treated with tamoxifen and exemestane. Nevertheless, certain QoL issues are treatment-specific and deserve attention by oncology health care providers. Also, the large number of patients who reported complaints of sexual functioning, fatigue, and insomnia in the QoL study was not mirrored by the reported adverse events related to these complaints in the main TEAM trial database. Although strictly observational, this large discrepancy between various QoL issues in the side study and the related adverse events recorded in the main trial stresses the need for careful inquiry by those seeing patients throughout the duration of endocrine treatment to optimize QoL and ensure adherence to treatment. Further investigation into an optimal reporting approach is warranted.
